# Rapidly improving acute respiratory distress syndrome in COVID-19: a multi-centre observational study

**DOI:** 10.1186/s12931-022-02015-8

**Published:** 2022-04-14

**Authors:** Evdokia Gavrielatou, Katerina Vaporidi, Vasiliki Tsolaki, Nikos Tserlikakis, George E. Zakynthinos, Eleni Papoutsi, Aikaterini Maragkuti, Athina G. Mantelou, Dimitrios Karayiannis, Zafeiria Mastora, Dimitris Georgopoulos, Epaminondas Zakynthinos, Christina Routsi, Spyros G. Zakynthinos, Edward J. Schenck, Anastasia Kotanidou, Ilias I. Siempos

**Affiliations:** 1 First Department of Critical Care Medicine and Pulmonary Services, Evangelismos Hospital, National and Kapodistrian University of Athens Medical School, 45-47 Ipsilantou Street, 10676 Athens, Greece; 2 Department of Intensive Care Medicine, University Hospital of Heraklion, Medical School University of Crete, Heraklion, Greece; 3 Critical Care Department, Faculty of Medicine, University Hospital of Larissa, University of Thessaly, Larissa, Greece; 4 Division of Pulmonary and Critical Care Medicine, Department of Medicine, New York-Presbyterian Hospital-Weill Cornell Medical Center, Weill Cornell Medicine, New York, NY USA

**Keywords:** Coronavirus, Acute respiratory failure, Trajectory, Pneumonia, Acute respiratory distress syndrome

## Abstract

**Background:**

Before the pandemic of coronavirus disease (COVID-19), rapidly improving acute respiratory distress syndrome (ARDS), mostly defined by early extubation, had been recognized as an increasingly prevalent subphenotype (making up 15–24% of all ARDS cases), associated with good prognosis (10% mortality in ARDSNet trials). We attempted to determine the prevalence and prognosis of rapidly improving ARDS and of persistent severe ARDS related to COVID-19.

**Methods:**

We included consecutive patients with COVID-19 receiving invasive mechanical ventilation in three intensive care units (ICU) during the second pandemic wave in Greece. We defined rapidly improving ARDS as extubation or a partial pressure of arterial oxygen to fraction of inspired oxygen ratio (PaO_2_:FiO_2_) greater than 300 on the first day following intubation. We defined persistent severe ARDS as PaO_2_:FiO_2_ of equal to or less than 100 on the second day following intubation.

**Results:**

A total of 280 intubated patients met criteria of ARDS with a median PaO_2_:FiO_2_ of 125.0 (interquartile range 93.0–161.0) on day of intubation, and overall ICU-mortality of 52.5% (ranging from 24.3 to 66.9% across the three participating sites). Prevalence of rapidly improving ARDS was 3.9% (11 of 280 patients); no extubation occurred on the first day following intubation. ICU-mortality of patients with rapidly improving ARDS was 54.5%. This low prevalence and high mortality rate of rapidly improving ARDS were consistent across participating sites. Prevalence of persistent severe ARDS was 12.1% and corresponding mortality was 82.4%.

**Conclusions:**

Rapidly improving ARDS was not prevalent and was not associated with good prognosis among patients with COVID-19. This is starkly different from what has been previously reported for patients with ARDS not related to COVID-19. Our results on both rapidly improving ARDS and persistent severe ARDS may contribute to our understanding of trajectory of ARDS and its association with prognosis in patients with COVID-19.

**Supplementary Information:**

The online version contains supplementary material available at 10.1186/s12931-022-02015-8.

## Background

Before the pandemic of coronavirus disease (COVID-19), researchers perceived acute respiratory distress syndrome (ARDS) as an heterogenous syndrome and identified several subphenotypes [1, 2]. One such subphenotype was based on the trajectory of hypoxemia [3] and was coined as rapidly improving ARDS [4]. Rapidly improving ARDS is present in patients who no longer meet the Berlin criteria or who are extubated within one day following intubation [4]. Prevalence of this subphenotype was up to 15% in recent therapeutic ARDSNet clinical trials [4], and even higher (reaching 24%) in a secondary analysis of the large observational LUNG SAFE study [5]. Mortality of patients with rapidly improving ARDS (approximately 10% in ARDSNet trials) was significantly lower (albeit still meaningful) than ARDS > 1 day [4]. Taken together, notwithstanding its unclear underlying pathobiology, rapidly improving ARDS is widely accepted as an increasingly prevalent subphenotype [6, 7], associated with better prognosis than ARDS > 1 day, and it is now taken into consideration by investigators performing randomized controlled trials [8–10].

Again, before COVID-19, it was also revealed based on the trajectory of hypoxemia that there is a subphenotype of persistent severe ARDS [11]. This is characterized by profound hypoxemia which persists for more than two days following intubation. Prevalence of this subphenotype was 15% in recent therapeutic ARDSNet clinical trials [11], and similar in a secondary analysis of the large observational LUNG SAFE study [12]. Mortality of patients with persistent severe ARDS was significantly higher than comparators [11]. Patients with persistent severe ARDS may share the landmark histopathological feature of ARDS, namely diffuse alveolar damage [13], and pose a big clinical challenge.

While trajectory-related subphenotypes (namely, rapidly improving ARDS and persistent severe ARDS) have been characterized in the pre-COVID era [4, 5, 11], little is known about the trajectory of ARDS in patients with COVID-19. Given that the trajectory of COVID-related ARDS is increasingly recognized as more clinically relevant than a single daily value of oxygenation [14, 15], we endeavoured to determine the prevalence and prognosis of rapidly improving ARDS and of persistent severe ARDS among intubated patients with COVID-19.

## Methods

### Study design

We performed a multi-center observational retrospective cohort study in patients with COVID-19 who received invasive mechanical ventilation during the second pandemic wave in Greece.

### Eligibility criteria

Adult patients (aged > 18 years) with polymerase chain reaction (PCR)-confirmed Severe Acute Respiratory Syndrome Coronavirus-2 (SARS-CoV-2) infection who received invasive mechanical ventilation due to hypoxemia [partial pressure of arterial oxygen to fraction of inspired oxygen ratio (PaO_2_:FiO_2_) equal to or less than 300], not fully explained by cardiac failure or fluid overload, and who had bilateral opacities in chest X-ray (i.e., patients who met the diagnostic criteria of ARDS according to the Berlin definition) [16] were considered eligible. Patients with PaO_2_:FiO_2_ more than 300 on the day of intubation were excluded. Eligible patients were consecutively recruited in academic ICUs at three tertiary hospitals in Athens (recruitment period: from October 21st, 2020 to March 8th, 2021), Crete (September 12th, 2020 to March 19th, 2021) and Larissa (August 7th, 2020 to June 17th, 2021). The three academic ICUs do not substantially differ in terms of care management; indeed, lung protective ventilation, conservative fluid and sedation vacation strategies are applied by full-time intensivists who are present around the clock. The Institutional Review Board at each participating study site, namely, Athens (Evangelismos Hospital: 116/31-03-2021), Crete (University Hospital of Heraklion: 567/07-07-2021) and Larissa (University Hospital: 53398/2020), approved of the data collection and waived the need of informed consent. The “Strengthening the Reporting of Observational Studies in Epidemiology” (STROBE) statement guidelines were applied (Additional file 1).

### Data collection and study groups

We collected data on demographics, comorbidities, usage (and its duration) of high-flow nasal oxygen and non-rebreather mask prior to intubation, usage of non-invasive mechanical ventilation prior to intubation, Sequential Organ Failure Assessment (SOFA) score on day of intubation [the respiratory component of SOFA was calculated after the intubation, while the remaining SOFA components (namely, coagulation, hepatic, cardiovascular, neurologic and renal) were calculated prior to intubation], ventilator settings and lung mechanics on the day of intubation as well on the first day and second day following intubation. We also gathered information on variables, which might affect trajectory of hypoxemia, such as level of positive end expiratory pressure (PEEP) and fluid balance along with general management of patients with ARDS (namely, steroids, prone positioning, neuromuscular blockade, inhaled nitric oxide and extracorporeal membrane oxygenation).

We categorized study patients into three groups. In accordance with previously reported definitions [4, 11], the “rapidly improving ARDS” group consisted of patients extubated or having a PaO_2_:FiO_2_ greater than 300 on the first day following intubation. The “persistent severe ARDS” group consisted of patients having a PaO_2_:FiO_2_ of equal to or less than 100 on the second day following intubation as well as of patients who were not alive on the second day following intubation. The remaining patients comprised the “intermediate” group.

### Study outcomes

Prevalence and ICU-mortality associated with rapidly improving ARDS and persistent severe ARDS among patients with COVID-19 were the primary outcomes of our study. Secondary outcomes were usage of vasopressors, vasopressor-free days, usage of continuous renal replacement therapy, continuous renal replacement therapy-free days, duration of mechanical ventilation among survivors, ventilator-free days and ICU-free days. Outcomes other than duration of mechanical ventilation were censored at day 28 following intubation. Patients discharged from ICU with unassisted breathing before 28 days considered to be alive at 28 days without needing vasopressors or continuous renal replacement therapy. Vasopressor-free days, continuous renal replacement therapy-free days, ventilator-free days and ICU-free days were calculated by the number of days in the first 28 days following intubation that a patient was alive and not receiving vasopressors, not receiving continuous renal replacement therapy, not on a ventilator or not in the ICU, respectively.

### Comparison with patients with ARDS not related to COVID-19

Two *post-hoc* comparisons were carried out. Firstly, prevalence of rapidly improving ARDS was compared between patients with ARDS related to COVID-19 (hospitalized in ICU of Crete during 2020–2021) and patients with ARDS related to influenza (hospitalized in ICU of Crete during 2017–2020). Secondly, outcomes of patients with rapidly improving ARDS due to pneumonia related to COVID-19 (included in our cohorts) were compared with those of patients with rapidly improving ARDS due to pneumonia not related to COVID-19. The latter group of patients with rapidly improving ARDS due to pneumonia not related to COVID-19 were derived from the Statins for Acutely Injured Lungs from Sepsis (SAILS) randomized controlled trial performed by the ARDSNet [17].

### Statistical analysis

No sample size calculation was performed a priori. Rather, the sample size was equal to the number of patients consecutively admitted in the participating ICUs during the study period. Continuous variables were presented as median with interquartile range (IQR) and compared using the Kruskal–Wallis test, with post-hoc pairwise comparisons using the Dunn–Bonferroni method. Categorical variables were presented as percentages and compared using the chi-squared or Fisher’s exact test, as appropriate. Kaplan Meier curve, with log-rank test for differences in survival functions between groups was applied for mortality. Α multiple variable regression analysis was carried out to isolate the contribution of age, modified SOFA score (i.e., total SOFA score minus the respiratory component of SOFA score) on day of intubation and change in PaO_2_:FiO_2_ from day of intubation to the first day following intubation (independent variables) to the ICU-mortality (dependent variable). To explore the potential “center effect”, an additional post-hoc multiple variable regression analysis (again with ICU-mortality as the dependent variable) was carried out, which used study site (namely, Athens, Crete or Larissa) as independent variable along with age and modified SOFA. Also, a multiple variable regression analysis was carried out to isolate the contribution of PaO_2_:FiO_2_, FiO_2_ and driving pressure on the day of intubation (independent variables derived from a previous relevant report of the pre-pandemic era) [11] to the development of persistent severe ARDS (dependent variable), while such an analysis for the development of rapidly improving ARDS was deemed meaningless due to small numbers. Missing data on outcomes were below 2% and completely at random according to Little’s MCAR test [18] and, therefore, a complete case analysis was performed. A p value less than 0.05 denoted statistical significance. Statistical analyses were performed using SPSS software ver. 25.0 (SPSS, Inc., Chicago, IL).

## Results

**Fig. 1:**
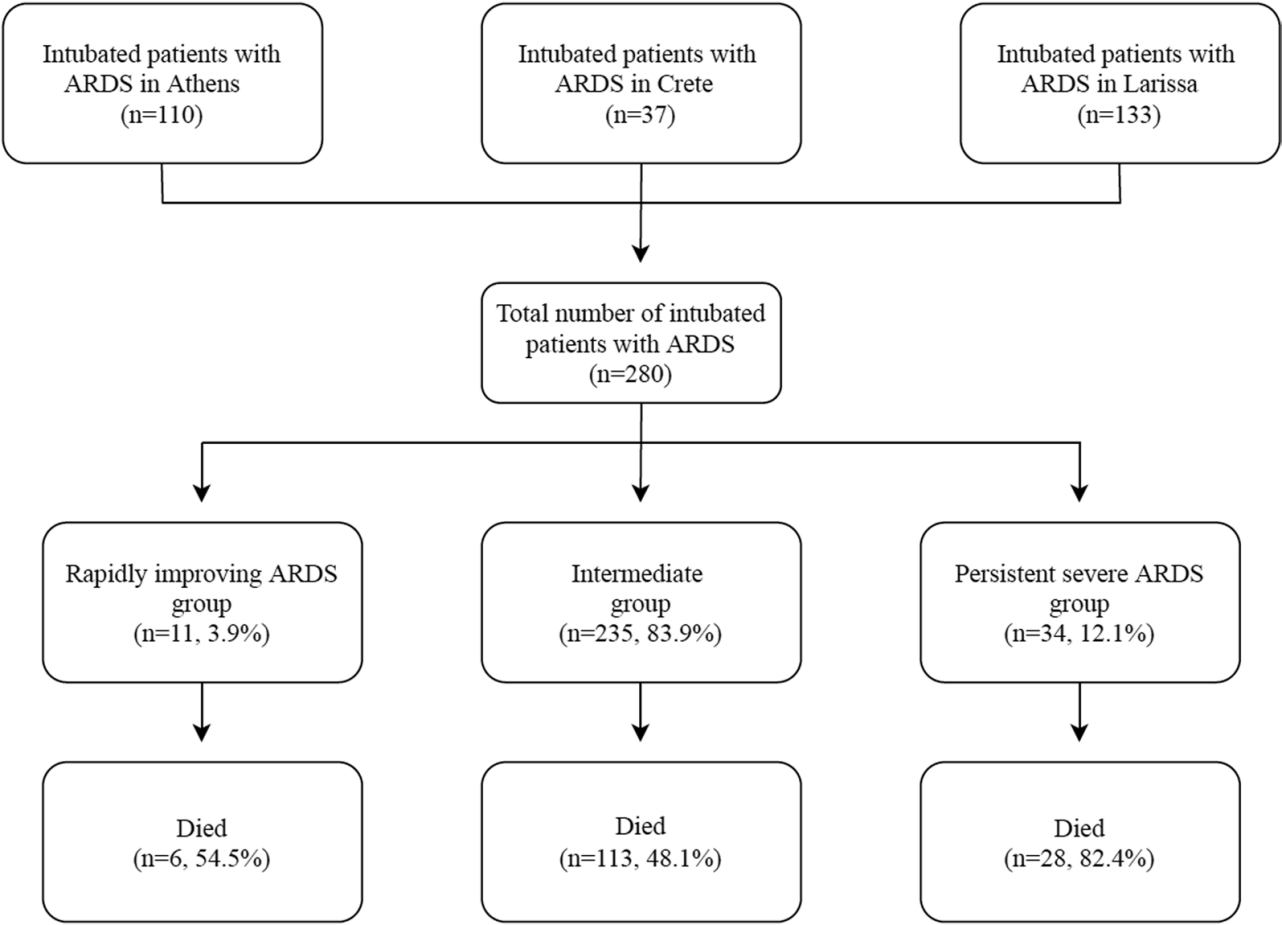
Distribution of included patients with acute respiratory distress (ARDS) across the three participating study sites. Out of the intubated patients with new coronavirus disease (COVID-19) who were hospitalized in the participating intensive care units during the study period, six patients (three from Athens and three from Larissa) did not meet the oxygenation criterion [i.e., they had partial pressure of arterial oxygen to fraction of inspired oxygen ratio (PaO_2_:FiO_2_) more than 300] of the Berlin definition of ARDS on the day of intubation and therefore were excluded from our study

A total of 280 patients with COVID-19, who received invasive mechanical ventilation and met criteria of ARDS, were included in our study. Figure 1 shows the distribution of patients across the three participating study sites.

### Differences between study sites

Additional file 1: Table S1 depicts baseline characteristics, lung mechanics and outcomes of included patients across the three participating sites (Athens, Crete and Larissa).

Regarding baseline characteristics and lung mechanics, there were no substantial differences between study sites in terms of demographics and comorbidities. Usage of high-flow nasal oxygen prior to intubation was more common in Athens (68.2%) and Crete (70.3%) compared to Larissa (43.4%). Median SOFA score on the day of intubation was 4.0 (IQR 4.0–5.0) for the combined cohort. Additional file 1: Fig. S1 shows the distribution of SOFA scores among all three study sites. Median PaO_2_:FiO_2_ on the day of intubation (calculated after the intubation) was 125.0 (93.0–161.0) for the combined cohort. Additional file 1: Fig. S2 shows the distribution of PaO_2_:FiO_2_ values among all three study sites. On the day of intubation, driving pressure was 12.0 (10.8–14.3) in Athens, 11.0 (10.0–12.0) in Crete and 13.0 (12.0–15.0) cmH_2_O in Larissa.

Regarding outcomes, ICU-mortality for the combined cohort was 52.5%, ranging from 24.3 (9 of 37 patients) in Crete to 44.5% (49 of 110) in Athens and 66.9% (89 of 133) in Larissa. A multiple variable regression analysis demonstrated that study site was an independent predictor of ICU-mortality after adjustment for age and modified SOFA score on day of intubation (Additional file 1: Table S2). There were differences across study sites in terms of usage of vasopressors and continuous renal replacement therapy-free days. There were no substantial differences across study sites in terms of vasopressor-free days, usage of continuous renal replacement therapy, duration of mechanical ventilation among survivors, ventilator-free days or ICU-free days (Additional file 1: Table S1).

### Baseline characteristics and lung mechanics of patients in each study group

Table 1 depicts baseline characteristics of patients included in each of the three study groups; namely, “rapidly improving ARDS” group, “intermediate” group and “persistent severe ARDS” group. There were no substantial differences between groups in terms of demographics, comorbidities, usage of high-flow nasal oxygen prior to intubation, and non-respiratory SOFA components scores on the day of intubation. Median SOFA score on the day of intubation was 4.0 (2.0–4.0) in the rapidly improving ARDS group, 4.0 (4.0–5.0) in the intermediate group and 4.5 (4.0–6.0) in the persistent severe ARDS group. Table 2 depicts lung mechanics of patients included in each of the three study groups. PaO_2_:FiO_2_ on the day of intubation was higher in the rapidly improving ARDS group [202.0 (162.0–227.0)] than in the intermediate group [125.0 (96.0–160.0)] and the persistent severe ARDS group [99.0 (73.0–150.0)]. Similarly, the corresponding median driving pressure values on the day of intubation was 12.0 (8.8–13.0), 12.0 (11.0–15.0) and 15.0 (12.8–17.3) cmH_2_O, respectively.

**Table 1:** Baseline characteristics of patients in each study group

	All (n = 280)	Rapidly improving ARDS group (n = 11)	Intermediate group (n = 235)	Persistent severe ARDS group (n = 34)	p value
Age, years	70.0 (61.0–76.0)	73.0 (46.0–78.0)	69.5 (61.0–76.0)	76.0 (58.5–80.0)	0.433
Female sex	88 (31.5)	5 (45.5)	72 (30.8)	11 (32.4)	0.588
Race					0.362
Caucasian	275 (98.6)	11 (100.0)	231 (98.7)	33 (97.1)	
Asian/Middle Eastern	2 (0.7)	0 (0.0)	2 (0.9)	0 (0.0)	
African	1 (0.4)	0 (0.0)	0 (0.0)	1 (2.9)	
Other	1 (0.4)	0 (0.0)	1 (0.4)	0 (0.0)	
Comorbidity	220 (78.6)	9 (81.8)	179 (76.2)	32 (94.1)	0.056
Chronic kidney disease	23 (8.2)	1 (9.1)	17 (7.2)	5 (14.7)	0.239
Chronic lung disease	40 (14.3)	1 (9.1)	35 (14.9)	4 (11.8)	0.931
Heart condition	76 (27.1)	4 (36.4)	59 (25.1)	13 (38.2)	0.214
Hypertension	171 (61.1)	5 (45.5)	140 (59.6)	26 (76.5)	0.093
Liver disease	3 (1.1)	1 (9.1)	1 (0.4)	1 (2.9)	0.032
Diabetes mellitus	71 (25.4)	3 (27.3)	57 (24.3)	11 (32.4)	0.591
Malignancy	25 (8.9)	2 (18.2)	20 (8.5)	3 (8.8)	0.454
SOFA score on the day of intubation	4.0 (4.0–5.0)	4.0 (2.0–4.0)	4.0 (4.0–5.0)	4.5 (4.0–6.0)	0.008^a,b^
Respiratory	4.0 (3.0–4.0)	3.0 (2.0–3.0)	4.0 (3.0–4.0)	4.0 (3.0–4.0)	< 0.001^a,b^
Coagulation	0.0 (0.0–0.0)	0.0 (0.0–0.0)	0.0 (0.0–0.0)	0.0 (0.0–0.0)	0.868
Hepatic	0.0 (0.0–0.0)	0.0 (0.0–0.0)	0.0 (0.0–0.0)	0.0 (0.0–0.0)	0.092
Cardiovascular	0.0 (0.0–0.0)	0.0 (0.0–0.0)	0.0 (0.0–0.0)	0.0 (0.0–0.0)	0.155
Neurologic	0.0 (0.0–0.0)	0.0 (0.0–0.0)	0.0 (0.0–0.0)	0.0 (0.0–0.0)	0.396
Renal	0.0 (0.0–0.0)	0.0 (0.0–1.0)	0.0 (0.0–0.0)	0.0 (0.0–1.0)	0.015^c^
Days from symptom onset to intubation	10.0 (6.0–13.0)	8.0 (4.0–10.0)	10.0 (6.0–13.0)	10.0 (4.5–15.0)	0.514
Usage of non-rebreather mask	116 (42.5)	6 (54.5)	98 (43.0)	12 (35.3)	0.497
Duration of non-rebreather mask, days	2.0 (1.0–3.0)	1.5 (1.0–2.3)	2.0 (1.0–3.0)	3.5 (1.3–5.0)	0.121
Usage of high-flow nasal oxygen	154 (57.2)	6 (60.0)	130 (57.5)	18 (54.5)	0.934
Duration of high-flow nasal oxygen, days	2.0 (1.0–5.0)	1.0 (1.0–3.0)	2.0 (1.0–5.0)	3.0 (1.8–4.0)	0.317
Usage of non-invasive mechanical ventilation	6 (2.5)	0 (0.0)	6 (3.0)	0 (0.0)	0.681
Severity of ARDS on the day of intubation					< 0.001
Mild	33 (11.8)	6 (54.5)	26 (11.1)	1 (3.0)	
Moderate	150 (53.8)	4 (36.4)	132 (56.2)	14 (42.4)	
Severe	96 (34.4)	1 (9.1)	77 (32.8)	18 (54.5)	
Management of ARDS after the intubation					
Steroids	209 (76.6)	10 (90.9)	178 (76.7)	21 (70.0)	0.371
Prone positioning	138 (50.4)	3 (33.3)	113 (48.9)	22 (64.7)	0.127
Neuromuscular blockade	280 (100.0)	11 (100.0)	235 (100.0)	34 (100.0)	–
Inhaled nitric oxide	0 (0.0)	0 (0.0)	0 (0.0)	0 (0.0)	–
ECMO	0 (0.0)	0 (0.0)	0 (0.0)	2 (5.9)	–

Data are presented as median (interquartile range) or number of patients (%)

Heart condition included congestive heart failure, coronary artery disease, and cardiomyopathies

Patients, who were intubated outside the intensive care unit, were admitted in the intensive care unit the same day

Non-invasive mechanical ventilation was delivered via face mask

Severity of ARDS was classified according to the Berlin definition

Administration of steroids was initiated prior to intubation

*n* number, *ARDS* acute respiratory distress syndrome, *SOFA* sequential organ failure assessment, *ECMO* extracorporeal membrane oxygenation

^a^Denotes statistical significance for the comparison between “rapidly improving ARDS” and “intermediate” groups

^b^Denotes statistical significance for the comparison between “rapidly improving ARDS” and “persistent severe ARDS” groups

^c^Denotes statistical significance for the comparison between “intermediate” and “persistent severe ARDS” groups

**Table 2:** Lung mechanics of patients in each study group

	All (n = 280)	Rapidly improving ARDS group (n = 11)	Intermediate group (n = 235)	Persistent severe ARDS group (n = 34)	p value
*Lung mechanics on the day of intubation*					
Ventilation mode					0.955
Volume control	238 (85.0)	9 (81.8)	200 (85.1)	29 (85.3)	
Pressure control	42 (15.0)	2 (18.2)	35 (14.9)	5 (14.7)	
Respiratory rate, bpm	25.0 (22.0–27.0)	22.0 (18.0–26.0)	25.0 (22.0–27.0)	25.0 (22.0–28.0)	0.136
Tidal volume, mL	450.0 (390.0–480.0)	430.0 (380.0–480.0)	450.0 (400.0–480.0)	425.0 (380.0–450.0)	0.067
Tidal volume/predicted body weight, mL/kg	6.4 (5.9–7.2)	7.2 (5.8–7.9)	6.5 (5.9–7.2)	6.0 (5.3–6.4)	0.021^c^
PEEPext, cmH_2_O	12.0 (10.0–14.0)	12.0 (10.0–14.0)	12.0 (10.0–14.0)	10.0 (10.0–13.0)	0.704
PEEPtotal, cmH_2_O	12.0 (10.0–14.0)	12.0 (10.5–14.0)	12.0 (10.0–14.0)	11.0 (10.0–13.0)	0.343
Pplateau, cmH_2_O	26.0 (22.0–28.0)	23.5 (20.0–26.3)	25.0 (22.0–28.0)	28.0 (25.0–30.0)	0.005^b,c^
Pdriving, cmH_2_O	13.0 (11.0–15.0)	12.0 (8.8–13.0)	12.0 (11.0–15.0)	15.0 (12.8–17.3)	0.001^b,c^
Compliance of respiratory system, mL/cmH_2_O	33.6 (28.9–40.9)	36.2 (30.5–49.0)	34.3 (29.2–41.7)	29.4 (22.3–33.6)	0.001^b,c^
FiO_2_	0.8 (0.6–1.0)	0.6 (0.5–1.0)	0.8 (0.6–1.0)	1.0 (0.7–1.0)	0.003^b,c^
PaO_2_, mmHg	88.0 (73.0–111.0)	101.0 (90.0–162.0)	88.0 (73.0–110.0)	84.0 (65.0–113.5)	0.164
PaO_2_:FiO_2_	125.0 (93.0–161.0)	202.0 (162.0–227.0)	125.0 (96.0–160.0)	99.0 (73.0–150.0)	< 0.001^a,b^
PaCO_2_, mmHg	46.0 (39.0–56.0)	45.0 (39.5–52.7)	46.0 (39.0–56.0)	50.0 (41.0–60.5)	0.421
*Lung mechanics on the first day following intubation*					
Ventilation mode					1.0
Volume control	274 (98.2)	11 (100.0)	229 (97.9)	34 (100.0)	
Pressure support	5 (1.8)	0 (0.0)	5 (2.1)	0 (0.0)	
FiO_2_	0.6 (0.5–0.7)	0.4 (0.4–0.5)	0.6 (0.5–0.7)	0.8 (0.7–0.9)	< 0.001^a−c^
PaO_2_, mmHg	90.0 (79.0–109.0)	141.0 (110.0–180.0)	90.0 (80.0–109.0)	85.0 (75.3–98.0)	< 0.001^a,b^
PaO_2_:FiO_2_	157.0 (127.0–201.8)	353.0 (314.0–368.0)	162.0 (134.0–200.0)	111.5 (91.5–135.8)	< 0.001^a−c^
*Lung mechanics on the second day following intubation*					
Positive fluid balance	231 (84.0)	9 (81.8)	194 (82.9)	28 (93.3)	0.304
Fluid balance, mL	1333.0 (420.0–2535.0)	1020.0 (737.0–1845.0)	1268.5 (350.0–2475.0)	2188.0 (1146.5–4067.3)	0.013^c^
Still intubated	271 (98.5)	10 (90.9)	231 (98.7)	30 (100.0)	0.206
Ventilation mode					0.210
Volume control	250 (94.0)	7 (87.5)	213 (93.4)	30 (100.0)	
Pressure support	16 (6.0)	1 (12.5)	15 (6.6)	0 (0.0)	
Respiratory rate, bpm	26.0 (23.0–28.0)	25.0 (21.5–28.0)	25.0 (22.0–28.0)	28.0 (25.0–31.3)	0.026^c^
Tidal volume, mL	450.0 (400.0–480.0)	480.0 (385.0–495.0)	450.0 (400.0–480.0)	420.0 (375.0–452.5)	0.175
Tidal volume/predicted body weight, mL/kg	6.5 (6.0–7.2)	7.4 (6.0–8.1)	6.5 (6.0–7.3)	6.3 (5.8–6.7)	0.192
PEEPext, cmH_2_O	11.0 (9.0–12.0)	11.0 (8.3–13.0)	11.0 (9.0–12.0)	11.0 (8.8–14.3)	0.658
PEEPtotal, cmH_2_O	11.0 (9.0–13.0)	11.0 (9.0–13.0)	11.0 (9.0–13.0)	11.0 (9.0–14.0)	0.889
Pplateau, cmH_2_O	24.0 (22.0–27.0)	22.5 (19.5–24.5)	24.0 (22.0–27.0)	28.0 (24.0–31.0)	< 0.001^b,c^
Pdriving, cmH_2_O	13.0 (11.0–15.0)	11.0 (9.0–13.0)	12.0 (10.0–14.0)	15.0 (14.0–19.0)	< 0.001^b,c^
Compliance of respiratory system, mL/cmH_2_O	33.3 (28.0–40.9)	37.4 (30.2–40.2)	34.1 (28.6–43.2)	26.4 (22.1–31.4)	< 0.001^c^
FiO_2_	0.6 (0.5–0.7)	0.4 (0.4–0.5)	0.5 (0.5–0.6)	0.9 (0.8–1.0)	< 0.001^a−c^
PaO_2_, mmHg	89.0 (77.0–102.3)	97.0 (85.9–154.0)	91.0 (79.5–104.5)	67.5 (61.5–84.0)	< 0.001^b,c^
PaO_2_:FiO_2_	167.0 (137.0–211.5)	243.0 (218.0–350.0)	169.5 (144.0–211.8)	82.0 (68.8–95.3)	< 0.001^a−c^
PaCO_2_, mmHg	45.0 (41.0–52.0)	40.0 (38.3–45.0)	45.5 (41.3–52.0)	47.5 (43.5–62.3)	0.008^b^

Data are presented as median (interquartile range) or number of patients (%)

*n* number, *ARDS* acute respiratory distress syndrome, *bpm* breaths per minute, *PEEP* positive end expiratory pressure, *Pplateau* plateau pressure, *Pdriving* driving pressure, *PaO*_*2*_ partial pressure of arterial oxygen, *FiO*_*2*_ fraction of inspired oxygen, *PaCO*_*2*_ partial pressure of arterial carbon dioxide

^a^Denotes statistical significance for the comparison between “rapidly improving ARDS” and “intermediate” groups

^b^Denotes statistical significance for the comparison between “rapidly improving ARDS” and “persistent severe ARDS” groups

^c^Denotes statistical significance for the comparison between “intermediate” and “persistent severe ARDS” groups

### Prevalence of rapidly improving ARDS and persistent severe ARDS

Of the 280 patients included in the study, only 11 (3.9%) had rapidly improving ARDS. Six (54.5%) of them had mild ARDS on the day of intubation. The median PaO_2_:FiO_2_ of patients with rapidly improving ARDS was 353.0 (314.0–368.0) and none was extubated on the first day following intubation. This low prevalence of rapidly improving ARDS was consistent across study sites; i.e., 1.8% (2 of 110 patients) in Athens, 5.4% (2 of 37) in Crete and 5.3% (7 of 133) in Larissa.

Of the 280 patients included in the study, 34 (12.1%) had persistent severe ARDS. Eighteen (54.5%) of them had severe ARDS on the day of intubation. The median PaO_2_:FiO_2_ of patients with persistent severe ARDS was 82.0 (68.8–95.3) on the second day following intubation. Prevalence of persistent severe ARDS was 10.9% (12 of 110 patients) in Athens, 2.7% (1 of 37) in Crete and 15.8% (21 of 133) in Larissa. Driving pressure on the day of intubation was independently associated with development of persistent severe ARDS (Additional file 1: Table S3).

### Outcomes of rapidly improving ARDS and persistent severe ARDS

**Fig. 2:**
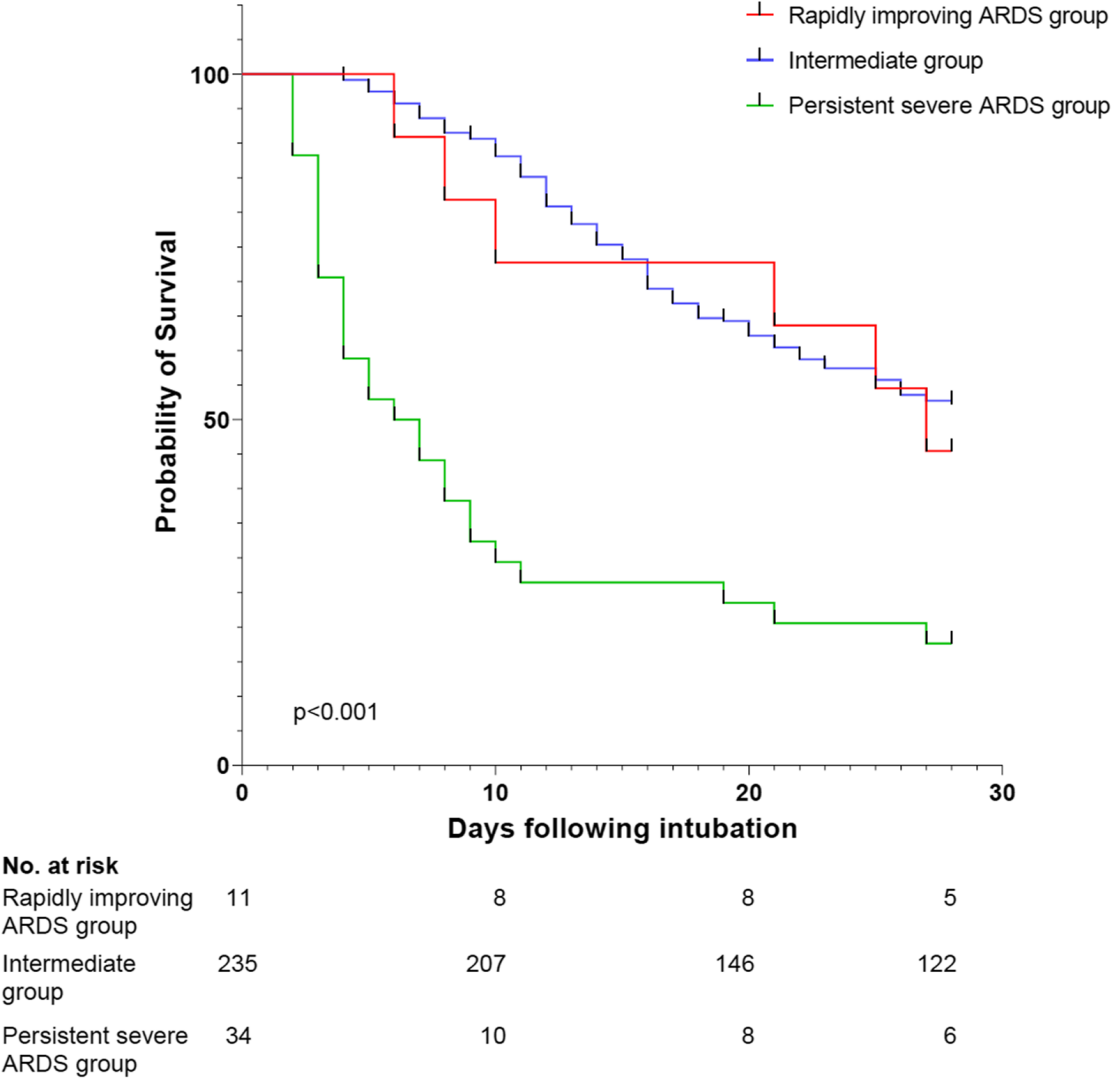
Kaplan–Meier curves of mortality for the three study groups. Differences in mortality between rapidly improving acute respiratory distress (ARDS) group, intermediate group and persistent severe ARDS group were assessed using the log-rank test. There was no statistically significant difference between rapidly improving ARDS group and intermediate group, while there were statistically significant differences between persistent severe ARDS group and rapidly improving ARDS group (p = 0.018) or intermediate group (p < 0.001). Patients discharged from the intensive care unit with unassisted breathing before 28 days considered to be alive at 28 days following intubation

Table 3 depicts outcomes of patients included in each of the three study groups. ICU-mortality was 54.5% (6 of 11 patients) in the rapidly improving ARDS group, 48.1% (113 of 235) in the intermediate group and 82.4% (28 of 34) in the persistent severe ARDS group. Figure 2 shows the Kaplan–Meier curves of mortality for the three study groups. ICU-mortality of patients in the rapidly improving ARDS group was not different from the intermediate group. Consistently, the multiple variable regression analysis demonstrated that change in PaO_2_:FiO_2_ from day of intubation to the first day following intubation was not an independent predictor of ICU-mortality even after adjustment for potential confounders, such as age and modified SOFA score on day of intubation (Additional file 1: Table S4). There were no substantial differences between groups in terms of usage of vasopressors, usage of continuous renal replacement therapy, duration of mechanical ventilation among survivors, ventilator-free days and ICU-free days. Patients in the rapidly improving ARDS group had more vasopressor-free days, while patients in the persistent severe ARDS group had fewer continuous renal replacement therapy-free days than comparators (Table 3).

**Table 3:** Outcomes of patients in each study group

	All (n = 280)	Rapidly improving ARDS group (n = 11)	Intermediate group (n = 235)	Persistent severe ARDS group (n = 34)	p value
Usage of vasopressors, n (%)	275 (99.3)	11 (100.0)	230 (99.1)	34 (100.0)	1.0
Vasopressor-free days, days	0.0 (0.0–12.0)	4.0 (1.0–24.0)	0.0 (0.0–14.3)	0.0 (0.0–0.0)	< 0.001^b,c^
Usage of continuous renal replacement therapy, n (%)	114 (41.2)	4 (36.4)	99 (42.7)	11 (32.4)	0.493
Continuous renal replacement therapy-free days, days	18.0 (6.0–28.0)	25.0 (6.0–28.0)	20.0 (8.0–28.0)	4.0 (2.8–10.3)	< 0.001^b,c^
Duration of mechanical ventilation among survivors, days	18.0 (8.3–34.8)	3.0 (2.3–5.3)	19.0 (9.0–35.5)	29.5 (10.0–34.5)	0.11
Ventilator-free days, days	0.0 (0.0–6.5)	0.0 (0.0–25.0)	0.0 (0.0–10.0)	0.0 (0.0–0.0)	0.011^b,c^
ICU-free days, days	0.0 (0.0–0.0)	0.0 (0.0–22.0)	0.0 (0.0–3.5)	0.0 (0.0–0.0)	0.016^b,c^
ICU-mortality, n (%)	147 (52.5)	6 (54.5)	113 (48.1)	28 (82.4)	0.001^b,c^

Data are presented as median (interquartile range) of number of patients (%)

Intermediate group includes two patients from Crete, who were transferred to another ICU on the 5th and 9th day following intubation, respectively. These patients were considered alive at day 28 following intubation. Persistent severe ARDS group includes 10 patients who were not alive on the second day following intubation

Outcomes other than duration of mechanical ventilation were censored at day 28 following intubation. Patients discharged from ICU with unassisted breathing before 28 days considered to be alive at 28 days without needing vasopressors or continuous renal replacement therapy. Vasopressor-free days, continuous renal replacement therapy-free days, ventilator-free days and ICU-free days were calculated by the number of days in the first 28 days following intubation that a patient was alive and not receiving vasopressors, not receiving continuous renal replacement therapy, not on a ventilator or not in the ICU, respectively

ICU-mortality was 42.4% (14 of 33) for patients with mild ARDS, 50.7% (76 of 150) for patients with moderate ARDS and 58.3% (56 of 96) for those with severe ARDS on the day of intubation

*n* number, *ARDS* acute respiratory distress syndrome, *ICU* intensive care unit

^a^Denotes statistical significance for the comparison between “rapidly improving ARDS” and “intermediate” groups

^b^Denotes statistical significance for the comparison between “rapidly improving ARDS” and “persistent severe ARDS” groups

^c^Denotes statistical significance for the comparison between “intermediate” and “persistent severe ARDS” groups

### Comparison with patients with ARDS not related to COVID-19

Prevalence of rapidly improving ARDS was 5.4% among patients with ARDS related to COVID-19 as opposed to 15.4% among patients with ARDS related to influenza (Additional file 1: Table S5). Outcomes, such as ventilator-free days (0.0 versus 27.0 days), ICU-free days (0.0 versus 24.0 days) and ICU-mortality (54.5% versus 12.9%), of patients with rapidly improving ARDS due to pneumonia related to COVID-19 were worse than those of patients with rapidly improving ARDS due to pneumonia not related to COVID-19 (Additional file 1: Table S6).

## Discussion

By incorporating data from 280 patients with COVID-19 who received invasive mechanical ventilation during the second pandemic wave in three academic ICUs, we found that rapidly improving ARDS was present in just 4% of included patients and it was associated with 55% mortality. Not even one patient was extubated on the first day following intubation. Less surprisingly, persistent severe ARDS was both prevalent (approximately 12% of cases) and associated with high mortality (approximately 82%) among patients with COVID-19.

We found that only one out of 25 intubated patients with COVID-19 had rapidly improving ARDS. Given that this prevalence was consistent across all three study sites, this finding seems robust. Notwithstanding its robustness, this finding is surprising because the approximately 4% prevalence of rapidly improving ARDS that we currently report is considerably lower than the up to 15% prevalence that it was previously reported in recent pre-pandemic ARDSNet randomized controlled trials [4]. One could attribute this difference in prevalence of rapidly improving ARDS between our observational study and previous ARDSNet trials to the well-documented differences between patients enrolled in randomized controlled trials (which have strict inclusion criteria) and those enrolled in observational studies. However, even the large pre-pandemic observational LUNG SAFE study reported a prevalence of rapidly improving ARDS of 24% [5], which is much higher than in our study. Thus, our robust finding of low prevalence of rapidly improving ARDS might not be sufficiently explained by our study design.

One therefore should seek for other potential explanations for our finding of the substantially lower prevalence of rapidly improving ARDS than previously reported. One could argue that previous reports might overestimate the prevalence of rapidly improving ARDS (and of ARDS generally) by including patients who might had alternate, easily reversible, noninflammatory causes of hypoxemia, such as atelectasis or cardiogenic pulmonary edema [19, 20]. Indeed, such reports included several patients with ARDS due to unknown risk factors, who might be more likely to experience rapid improvement of their syndrome compared to patients with ARDS due to known risk factors [21]. The fact that COVID-related ARDS has a known risk factor and specifically the fact that this risk factor is pulmonary infection (a “direct” risk factor), which has been identified as the factor least likely to be associated with rapidly improving ARDS [5], might explain our observed low prevalence of rapidly improving ARDS. That being said, even when we calculated the prevalence of rapidly improving ARDS among patients with ARDS related to influenza and hospitalized in one participating study site (Crete), we found it as high as 15.4% which is closer to that previously reported [4, 5] rather than it of COVID-related ARDS. Therefore, presence of a “direct” risk factor (viral pneumonia) might not fully explain the low prevalence of rapidly improving ARDS in our study.

Beyond the risk factor, one could hypothesize an association between the potential of patient self-inflicted lung injury due to prolonged usage of high-flow nasal oxygen and/or non-rebreather mask prior to intubation and subsequent low prevalence of rapidly improving ARDS [22]. However, in the present study, patients with rapidly improving ARDS did not substantially differ from comparators in terms of usage and duration of high-flow nasal oxygen and/or non-rebreather mask. Besides, it is unclear whether a trial of high-flow nasal oxygen might deteriorate outcomes of patients with COVID-19 [23]. On the other hand, although one could think that rapidly improving ARDS is not essentially different from mild ARDS, we found that almost half of patients with rapidly improving ARDS had moderate or severe (rather than mild) ARDS on the day of intubation (Table 1). Taken together, usage of high-flow nasal oxygen prior to intubation and severity of ARDS (i.e., categorization as mild ARDS according to the Berlin definition) [16] on the day of intubation might not fully explain the low prevalence of rapidly improving ARDS in our study.

We found that mortality of patients with rapidly improving ARDS was as high as 55%. This is surprising as it is considerably higher than that reported in the literature before the pandemic (10% mortality in ARDSNet trials) [4]. One could attribute this surprising finding to the fact that pre-pandemic literature usually included a heterogenous population of patients with ARDS due to various risk factors (or even patients with ARDS due to unknown risk factors) with varying attributable mortality [24], whereas the population of patients with ARDS due to COVID-19 may be more homogenous having viral pneumonia as risk factor associated with considerable mortality. However, even when we compared outcomes of patients with rapidly improving ARDS due to pneumonia related to COVID-19 (included in our cohorts) with those of patients with rapidly improving ARDS due to pneumonia not related to COVID-19 (included in the SAILS ARDSNet trial) [17], we found them worse (keeping in mind though that differences in age or comorbidities between comparators might partially explain this finding). That being said, one should be cautious not to infer that trajectory of hypoxemia in COVID-related ARDS does not predict mortality. A recent observational study from Italy reported that progressive increases in PaO_2_:FiO_2_ showed a higher association with survival compared to a single value of PaO_2_:FiO_2_ on the day of intubation [14]. However, in the Italian study, the change of PaO_2_:FiO_2_ was assessed throughout the ICU stay rather than from the day of intubation to the first day following intubation [14]. By combining the results of the Italian and our study, one could deduce that just one day may be a short time period to determine whether improvements in arterial blood gases will translate into lower mortality of patients with ARDS due to COVID-19. This deduction might be supported by our multiple variable regression analysis showing that modified SOFA score (which included SOFA components other than respiratory) on the day of intubation was a stronger predictor of mortality than change in PaO_2_:FiO_2_ from day of intubation to the first day following intubation.

On the other hand, our findings regarding persistent severe ARDS seem anticipated. The prevalence of persistent severe ARDS seems similar to non-COVID ARDS [11] (or even non-COVID acute hypoxemic respiratory failure) [25] and so is the mortality. This is also in line with findings of a recent observational study from the United Kingdom, which reported that refractory hypoxemia remains a major determinant of mortality in the COVID-19 era [15].

Our study has limitations. Firstly, although experts recently suggested that the Berlin definition of ARDS should be broadened to include patients treated with high-flow nasal oxygen of at least 30 L/min [26], we chose to focus on patients who received invasive mechanical ventilation. Our choice allowed us for directly comparing our findings with previous reports on rapidly improving ARDS, which also focused on patients receiving invasive mechanical ventilation [4, 5]. Secondly, we lacked data on the management of patients prior to intubation (such as usage of immunomodulatory agents and delays between onset of symptoms and hospitalization or initiation of first-line ventilatory support) as well as their complications (such as infections) after intubation. However, a fairly complicated disease course of the included patients could be safely presumed given their prolonged ICU stay, as noted by a median of zero ICU-free days in Table 3 and Additional file 1: Table S1. Surprisingly, a median of zero ICU-free days and zero ventilator-free days was the case even for patients with rapidly improving ARDS (although those who eventually survived had a median duration of mechanical ventilation of three days) indicating that their initial improvement was not sustained; indeed, their median oxygenation on the second day following intubation was worse than the day before (PaO_2_:FiO_2,_ 243 versus 353; Table 2). Taken together, one could deduce that just one day may not enough to determine whether improvement in oxygenation will translate into shorter ICU stay or shorter ventilatory support of patients with ARDS due to COVID-19.

Thirdly, our study included patients from three participating ICUs located in central (Athens), southern (Crete) and northern (Larissa) Greece with different health-care system strains during the study period. Health-care system strain has been recognized as a major determinant of outcomes of critically ill patients with COVID-19 [27] and might explain why ICU-mortality was lower in one study site (Crete, enrolling 37 patients) than in another (Larissa, 133 patients); differences which persisted even after adjustment for confounders, such as age and modified SOFA score on day of intubation (Additional file 1: Table S2). However, the fact that, despite their differences (reflected in different ICU-mortality), all three study sites reported similar prevalence and mortality rates of rapidly improving ARDS might enhance the robustness of our finding.

Finally, one could consider our reported ICU-mortality of above 50% for the combined cohort (and specifically above 80% among patients with persistent severe ARDS) as high. Indeed, although we had reported low mortality rate of intubated patients with COVID-19 in Greek ICUs during the first wave [28], we noticed an increase during the second wave [29]. During the second wave, patient load was high enough to put the Greek healthcare system (which admittedly had few reserves after years of underfunding due to a precedent long financial crisis) [30] at intermediate stress levels, when the availability of care was not nominally restricted but still adversely affected outcomes [31]. Interestingly, other countries, such as the United States [32] and Germany [33], also reported similar mortality rates (i.e., above 50%) of mechanically ventilated patients during the second wave.

## Conclusion

In conclusion, rapidly improving ARDS was not prevalent and was not associated with any survival benefit among patients with COVID-19. This is starkly different from what has been previously reported for patients with ARDS not related to COVID-19. Our results on both rapidly improving and persistent severe ARDS may contribute to our understanding of trajectory of ARDS and its association with prognosis in patients with COVID-19. Specifically, our results may inform discussions with families about prognosis as clinicians could be aware that just one day may not be enough to determine whether improvement in oxygenation will translate into lower mortality or shorter ICU stay of patients with ARDS due to COVID-19.

## Supplementary Information

**Additional file 1: Table 1.** Baseline characteristics, lung mechanics and outcomes of included patients across the three participating study sites. **Figure S1.** Distribution of scores of Sequential Organ Failure Assessment (SOFA) on the day of intubation among the three study sites. **Figure S2.** Distribution of partial pressure of arterial oxygen to fraction of inspired oxygen ratio (PaO_2_:FiO_2_) values on the day of intubation (calculated after intubation) among the three study sites. **Table S2.** Univariable and multiple variable regression analysis to isolate the contribution of age, modified SOFA score on the day of intubation and study site (independent variables) to the ICU-mortality (dependent variable). **Table S3.** Univariable and multiple variable regression analysis to isolate the contribution of PaO_2_:FiO_2_, FiO_2_ and driving pressure on the day of intubation (independent variables) to the development of persistent severe ARDS (dependent variable). **Table S4.** Univariable and multiple variable regression analysis to isolate the contribution of age, modified SOFA score on the day of intubation and change in PaO_2_:FiO_2_ from day of intubation to the first day following intubation (independent variables) to the ICU-mortality (dependent variable). **Table S5.** Characteristics and outcomes of patients hospitalized in Crete with ARDS related to COVID-19 versus influenza. **Table S6.** Characteristics and outcomes of patients with rapidly improving ARDS due to pneumonia related or not to COVID-19.

## Data Availability

The datasets used and/or analyzed during the current study are available from the corresponding author on reasonable request.
