# Supplementary material for: Rapidly improving acute respiratory distress syndrome in COVID-19: a multi-centre observational study
Source: Respir Res. 2022 Apr 14;23:94. doi: 10.1186/s12931-022-02015-8 (PMC9008400; doi:10.1186/s12931-022-02015-8)
Supplement: Supplementary file 1 — Additional file 1: Table 1. Baseline characteristics, lung mechanics and outcomes of included patients across the three participating study sites. Figure S1. Distribution of scores of Sequential Organ Failure Assessment (SOFA) on the day of intubation among the three study sites. Figure S2. Distribution of partial pressure of arterial oxygen to fraction of inspired oxygen ratio (PaO2:FiO2) values on the day of intubation (calculated after intubation) among the three study sites. Table S2. Univariable and multiple variable regression analysis to isolate the contribution of age, modified SOFA score on the day of intubation and study site (independent variables) to the ICU-mortality (dependent variable). Table S3. Univariable and multiple variable regression analysis to isolate the contribution of PaO2:FiO2, FiO2 and driving pressure on the day of intubation (independent variables) to the development of persistent severe ARDS (dependent variable). Table S4. Univariable and multiple variable regression analysis to isolate the contribution of age, modified SOFA score on the day of intubation and change in PaO2:FiO2 from day of intubation to the first day following intubation (independent variables) to the ICU-mortality (dependent variable). Table S5. Characteristics and outcomes of patients hospitalized in Crete with ARDS related to COVID-19 versus influenza. Table S6. Characteristics and outcomes of patients with rapidly improving ARDS due to pneumonia related or not to COVID-19. [file 12931_2022_2015_MOESM1_ESM.docx]

**Supplemental Material**

**Title:** Rapidly improving acute respiratory distress syndrome in COVID-19: a multi-centre observational study

**Authors:** Evdokia Gavrielatou, Katerina Vaporidi, Vasiliki Tsolaki, Nikos Tserlikakis, George E. Zakynthinos, Eleni Papoutsi, Aikaterini Maragkuti, Athina G. Mantelou, Dimitrios Karayiannis, Zafeiria Mastora, Dimitris Georgopoulos, Epaminondas Zakynthinos, Christina Routsi, Spyros G. Zakynthinos, Edward J. Schenck, Anastasia Kotanidou, Ilias I. Siempos

**“Strengthening the Reporting of Observational Studies in Epidemiology” (STROBE) Checklist**

|  | Item No. | Recommendation | Page  No. |
| --- | --- | --- | --- |
| **Title and abstract** | 1 | (*a*) Indicate the study’s design with a commonly used term in the title or the abstract | 1 |
|  |  | (*b*) Provide in the abstract an informative and balanced summary of what was done and what was found | 2, 3 |
| Introduction | | | |
| Background/rationale | 2 | Explain the scientific background and rationale for the investigation being reported | 4, 5 |
| Objectives | 3 | State specific objectives, including any prespecified hypotheses | 5 |
| Methods | | | |
| Study design | 4 | Present key elements of study design early in the paper | 5 |
| Setting | 5 | Describe the setting, locations, and relevant dates, including periods of recruitment, exposure, follow-up, and data collection | 5, 6 |
| Participants | 6 | (*a*) *Cohort study*—Give the eligibility criteria, and the sources and methods of selection of participants. Describe methods of follow-up  *Case-control study*—Give the eligibility criteria, and the sources and methods of case ascertainment and control selection. Give the rationale for the choice of cases and controls  *Cross-sectional study*—Give the eligibility criteria, and the sources and methods of selection of participants | 5, 6 |
|  |  | (*b*) *Cohort study*—For matched studies, give matching criteria and number of exposed and unexposed  *Case-control study*—For matched studies, give matching criteria and the number of controls per case |  |
| Variables | 7 | Clearly define all outcomes, exposures, predictors, potential confounders, and effect modifiers. Give diagnostic criteria, if applicable | 6, 7 |
| Data sources/ measurement | 8* | For each variable of interest, give sources of data and details of methods of assessment (measurement). Describe comparability of assessment methods if there is more than one group | 6, 7 |
| Bias | 9 | Describe any efforts to address potential sources of bias | 7 |
| Study size | 10 | Explain how the study size was arrived at | 8 |

| Quantitative variables | 11 | Explain how quantitative variables were handled in the analyses. If applicable, describe which groupings were chosen and why | 8, 9 | |
| --- | --- | --- | --- | --- |
| Statistical methods | 12 | (*a*) Describe all statistical methods, including those used to control for confounding | | 8, 9 |
|  |  | (*b*) Describe any methods used to examine subgroups and interactions | | 8 |
|  |  | (*c*) Explain how missing data were addressed | | 8 |
|  |  | (*d*) *Cohort study*—If applicable, explain how loss to follow-up was addressed  *Case-control study*—If applicable, explain how matching of cases and controls was addressed  *Cross-sectional study*—If applicable, describe analytical methods taking account of sampling strategy | | 8 |
|  |  | (*e*) Describe any sensitivity analyses | | 8 |
|  |  | **Results** | |  |
| Participants | 13* | (a) Report numbers of individuals at each stage of study—eg numbers potentially eligible, examined for eligibility, confirmed eligible, included in the study, completing follow-up, and analysed | | 9 |
|  |  | (b) Give reasons for non-participation at each stage | | 9 |
|  |  | (c) Consider use of a flow diagram | | 9 |
| Descriptive data | 14* | (a) Give characteristics of study participants (eg demographic, clinical, social) and information on exposures and potential confounders | | 9-11 |
|  |  | (b) Indicate number of participants with missing data for each variable of interest | | 8 |
|  |  | (c) *Cohort study*—Summarise follow-up time (eg, average and total amount) | |  |
| Outcome data | 15* | *Cohort study*—Report numbers of outcome events or summary measures over time | | 10, 11 |
|  |  | *Case-control study—*Report numbers in each exposure category, or summary measures of exposure | |  |
|  |  | *Cross-sectional study—*Report numbers of outcome events or summary measures | |  |
| Main results | 16 | (*a*) Give unadjusted estimates and, if applicable, confounder-adjusted estimates and their precision (eg, 95% confidence interval). Make clear which confounders were adjusted for and why they were included | | 10-12 |
|  |  | (*b*) Report category boundaries when continuous variables were categorized | |  |
|  |  | (*c*) If relevant, consider translating estimates of relative risk into absolute risk for a meaningful time period | |  |

| Other analyses | 17 | Report other analyses done—eg analyses of subgroups and interactions, and sensitivity analyses | 12 |
| --- | --- | --- | --- |
|  |  | **Discussion** |  |
| Key results | 18 | Summarise key results with reference to study objectives | 12 |
| Limitations | 19 | Discuss limitations of the study, taking into account sources of potential bias or imprecision. Discuss both direction and magnitude of any potential bias | 15-17 |
| Interpretation | 20 | Give a cautious overall interpretation of results considering objectives, limitations, multiplicity of analyses, results from similar studies, and other relevant evidence | 12-14 |
| Generalisability | 21 | Discuss the generalisability (external validity) of the study results | 12 |
| Other information | |  |  |
| Funding | 22 | Give the source of funding and the role of the funders for the present study and, if applicable, for the original study on which the present article is based | 18 |

| **e-Table 1. Baseline characteristics, lung mechanics and outcomes of included patients across the three participating study sites.** | | | | | |
| --- | --- | --- | --- | --- | --- |
|  | **All (n=280)** | **Athens (n=110)** | **Crete (n=37)** | **Larissa  (n=133)** | **P value** |
| Age, years | 70.0 (61.0-76.0) | 69.0 (55.0-76.0) | 73.0 (64.0-78.5) | 70.0 (62.0-76.0) | 0.091 |
| Female sex | 88 (31.5) | 27 (24.8) | 15 (40.5) | 46 (34.6) | 0.118 |
| Race |  |  |  |  | 0.508 |
| Caucasian | 275 (98.6) | 107 (97.3) | 36 (100.0) | 132 (99.2) |  |
| Asian/Middle Eastern | 2 (0.7) | 2 (1.8) | 0 (0.0) | 0 (0.0) |  |
| African | 1 (0.4) | 1 (0.9) | 0 (0.0) | 0 (0.0) |  |
| Other | 1 (0.4) | 0 (0.0) | 0 (0.0) | 1 (0.8) |  |
| Comorbidity | 220 (78.6) | 81 (73.6) | 30 (81.1) | 109 (82.0) | 0.268 |
| Chronic kidney disease | 23 (8.2) | 14 (12.7) | 4 (10.8) | 5 (3.8) | 0.033 |
| Chronic lung disease | 40 (14.3) | 17 (15.5) | 7 (18.9) | 16 (12.0) | 0.516 |
| Heart condition | 76 (27.1) | 28 (25.5) | 11 (29.7) | 37 (27.8) | 0.854 |
| Hypertension | 171 (61.1) | 60 (54.5) | 23 (62.2) | 88 (66.2) | 0.179 |
| Liver disease | 3 (1.1) | 0 (0.0) | 0 (0.0) | 3 (2.3) | 0.363 |
| Diabetes mellitus | 71 (25.4) | 24 (21.8) | 12 (32.4) | 35 (26.3) | 0.412 |
| Malignancy | 25 (8.9) | 8 (7.3) | 5 (13.5) | 12 (9.0) | 0.515 |
| SOFA score on the day of intubation | 4.0 (4.0-5.0) | 4.0 (4.0-5.0) | 4.0 (4.0-5.0) | 4.0 (3.0-5.0) | 0.050 |
| Respiratory | 4.0 (3.0-4.0) | 4.0 (4.0-4.0) | 4.0 (3.0-4.0) | 4.0 (3.0-4.0) | <0.001 |
| Coagulation | 0.0 (0.0-0.0) | 0.0 (0.0-0.0) | 0.0 (0.0-0.0) | 0.0 (0.0-0.0) | 0.550 |
| Hepatic | 0.0 (0.0-0.0) | 0.0 (0.0-0.0) | 0.0 (0.0-0.0) | 0.0 (0.0-0.0) | 0.203 |
| Cardiovascular | 0.0 (0.0-0.0) | 0.0 (0.0-0.0) | 0.0 (0.0-0.0) | 0.0 (0.0-0.0) | 0.040 |
| Neurologic | 0.0 (0.0-0.0) | 0.0 (0.0-0.0) | 0.0 (0.0-1.0) | 0.0 (0.0-0.0) | <0.001 |
| Renal | 0.0 (0.0-0.0) | 0.0 (0.0-0.0) | 0.0 (0.0-0.5) | 0.0 (0.0-0.5) | 0.981 |
| Days from symptom onset to intubation | 10.0 (6.0-13.0) | 7.0 (4.0-11.0) | 9.5 (5.8-13.0) | 10.0 (8.0-15.0) | <0.001 |
| Usage of high-flow nasal oxygen | 154 (57.2) | 75 (68.2) | 26 (70.3) | 53 (43.4) | <0.001 |
| Duration of high-flow nasal oxygen, days | 2.0 (1.0-5.0) | 2.0 (1.0-5.0) | 2.0 (1.0-5.0) | 3.0 (2.0-3.5) | 0.375 |
| Usage of non-rebreather mask | 116 (42.5) | 22 (20.0) | 13 (37.1) | 81 (63.3) | <0.001 |
| Duration of non-rebreather mask, days | 2.0 (1.0-3.0) | 2.0 (1.0-3.0) | 1.0 (1.0-3.0) | 2.0 (1.0-3.5) | 0.433 |
| Usage of non-invasive mechanical ventilation | 6 (2.5) | 2 (1.8) | NA | 4 (3.0) | 0.693 |
| Severity of ARDS on the day of intubation |  |  |  |  | <0.001 |
| Mild | 33 (11.8) | 15 (13.6) | 6 (16.7) | 12 (9.0) |  |
| Moderate | 150 (53.8) | 70 (63.6) | 26 (72.2) | 54 (40.6) |  |
| Severe | 96 (34.4) | 25 (22.7) | 4 (11.1) | 67 (50.4) |  |
| **Management of ARDS after the intubation** |  |  |  |  |  |
| Steroids | 209 (76.6) | 81 (75.0) | 36 (100.0) | 92 (71.3) | 0.001 |
| Prone positioning | 138 (50.4) | 26 (23.9) | 23 (67.6) | 89 (67.9) | <0.001 |
| Neuromuscular blockade | 280 (100.0) | 110 (100.0) | 37 (100.0) | 133 (100.0) | - |
| Inhaled nitric oxide | 0 (0.0) | 0 (0.0) | 0 (0.0) | 0 (0.0) | - |
| ECMO | 0 (0.0) | 0 (0.0) | 0 (0.0) | 0 (0.0) | - |
| **Lung mechanics on the day of intubation** |  |  |  |  |  |
| Ventilation mode |  |  |  |  | <0.001 |
| Volume Control | 238 (85.0) | 68 (61.8) | 37 (100.0) | 133 (100.0) |  |
| Pressure Control | 42 (15.0) | 42 (38.2) | 0 (0.0) | 0 (0.0) |  |
| Respiratory rate, bpm | 25.0 (22.0-27.0) | 25.0 (22.0-28.0) | 27.5 (26.0-29.0) | 24.0 (20.0-26.0) | <0.001 |
| Tidal volume, mL | 450.0 (390.0-480.0) | 480.0 (440.0-500.0) | 380.0 (370.0-415.0) | 430.0 (385.0-475.0) | <0.001 |
| Tidal volume/predicted body weight, mL/kg | 6.4 (5.9-7.2) | 6.8 (6.4-7.9) | 6.1 (5.6-7.2) | 6.4 (5.9-7.2) | 0.072 |
| PEEPext, cmH_2_O | 12.0 (10.0-14.0) | 12.0 (10.0-13.0) | 14.0 (10.0-16.0) | 12.0 (10.0-14.0) | 0.013 |
| PEEPtotal, cmH_2_O | 12.0 (10.0-14.0) | 13.0 (10.0-14.0) | 14.0 (11.0-16.0) | 12.0 (10.0-14.0) | 0.003 |
| Pplateau, cmH_2_O | 26.0 (22.0-28.0) | 25.0 (23.0-27.8) | 26.0 (22.0-27.0) | 26.0 (22.0-29.0) | 0.457 |
| Pdriving, cmH_2_O | 13.0 (11.0-15.0) | 12.0 (10.8-14.3) | 11.0 (10.0-12.0) | 13.0 (12.0-15.0) | <0.001 |
| Compliance of respiratory system, mL/cmH_2_O | 33.6 (28.9-40.9) | 37.8 (30.0-45.1) | 33.9 (30.8-38.1) | 31.3 (26.4-39.7) | 0.001 |
| FiO_2_ | 0.8 (0.6-1.0) | 0.9 (0.7-1.0) | 0.7 (0.6-0.7) | 0.7 (0.6-1.0) | <0.001 |
| PaO_2_, mmHg | 88.0 (73.0-111.0) | 108.0 (87.0-135.3) | 84.5 (73.8-93.4) | 80.0 (65.5-96.0) | <0.001 |
| PaO_2_:FiO_2_ | 125.0 (93.0-161.0) | 140.0 (106.8-167.5) | 136.5 (114.0-168.3) | 100.0 (80.0-151.5) | <0.001 |
| PaCO_2_, mmHg | 46.0 (39.0-56.0) | 47.0 (41.0-56.3) | 40.5 (36.0-46.7) | 48.0 (39.0-57.0) | 0.002 |
| **Lung mechanics on the first day following intubation** |  |  |  |  |  |
| Ventilation mode |  |  |  |  | 0.052 |
| Volume Control | 274 (98.2) | 105 (96.3) | 36 (97.3) | 133 (100.0) |  |
| Pressure Support | 5 (1.8) | 4 (3.7) | 1 (2.7) | 0 (0.0) |  |
| FiO_2_ | 0.6 (0.5-0.7) | 0.6 (0.6-0.8) | 0.5 (0.4-0.6) | 0.6 (0.5-0.7) | <0.001 |
| PaO_2_, mmHg | 90.0 (79.0-109.0) | 103.0 (85.8-120.0) | 86.5 (74.8-94.5) | 86.0 (77.0-99.5) | <0.001 |
| PaO_2_:FiO_2_ | 157.0 (127.0-201.8) | 158.0 (128.5-196.5) | 177.0 (133.5-221.5) | 153.0 (118.0-201.5) | 0.192 |
| Rapidly improving ARDS | 11 (3.9) | 2 (1.8) | 2 (5.4) | 7 (5.3) | 0.294 |
| **Lung mechanics on the second day following intubation** |  |  |  |  |  |
| Positive fluid balance | 231 (84.0) | 84 (77.8) | 25 (69.4) | 122 (93.1) | <0.001 |
| Fluid balance, mL | 1333.0 (420.0-2535.0) | 1100.0 (40.0-2210.0) | 1383.5 (-334.8-2818.8) | 1545.0 (834.3-2659.5) | 0.028 |
| Still intubated | 271 (98.5) | 106 (97.2) | 36 (100.0) | 129 (99.2) | 0.362 |
| Ventilation mode |  |  |  |  | 0.008 |
| Volume Control | 250 (94.0) | 90 (88.2) | 33 (97.1) | 127 (97.7) |  |
| Pressure Support | 16 (6.0) | 12 (11.8) | 1 (2.9) | 3 (2.3) |  |
| Respiratory rate, bpm | 26.0 (23.0-28.0) | 28.0 (24.0-30.0) | 27.5 (24.0-30.0) | 25.0 (22.0-27.0) | <0.001 |
| Tidal volume, mL | 450.0 (400.0-480.0) | 475.0 (420.0-500.0) | 380.0 (352.5-430.0) | 450.0 (400.0-480.0) | <0.001 |
| Tidal volume/predicted body weight, mL/kg | 6.5 (6.0-7.2) | 6.9 (6.4-8.2) | 6.4 (5.9-6.9) | 6.4 (5.9-7.3) | 0.058 |
| PEEPext, cmH_2_O | 11.0 (9.0-12.0) | 11.0 (9.3-12.0) | 12.0 (11.0-15.0) | 10.0 (9.0-12.0) | <0.001 |
| PEEPtotal, cmH_2_O | 11.0 (9.0-13.0) | 12.0 (10.0-14.0) | 13.0 (11.0-15.0) | 10.0 (9.0-12.0) | <0.001 |
| Pplateau, cmH_2_O | 24.0 (22.0-27.0) | 24.0 (22.0-27.0) | 24.0 (21.5-27.0) | 24.0 (22.0-27.0) | 0.895 |
| Pdriving, cmH_2_O | 13.0 (11.0-15.0) | 12.0 (10.0-14.0) | 11.0 (10.0-12.0) | 13.0 (12.0-16.0) | <0.001 |
| Compliance of respiratory system, mL/cmH_2_O | 33.3 (28.0-40.9) | 34.3 (30.7-45.7) | 35.4 (31.7-40.5) | 31.9 (26.4-40.0) | 0.017 |
| FiO_2_ | 0.6 (0.5-0.7) | 0.6 (0.5-0.7) | 0.5 (0.4-0.6) | 0.5 (0.5-0.6) | <0.001 |
| PaO_2_, mmHg | 89.0 (77.0-102.3) | 98.0 (87.0-120.0) | 80.0 (72.0-92.6) | 85.0 (73.8-95.0) | <0.001 |
| PaO_2_:FiO_2_ | 167.0 (137.0-211.5) | 167.0 (138.5-209.0) | 181.5 (140.8-210.0) | 164.0 (130.5-213.0) | 0.572 |
| PaCO_2_, mmHg | 45.0 (41.0-52.0) | 47.0 (43.0-53.8) | 40.5 (36.9-44.0) | 47.0 (42.0-53.0) | <0.001 |
| Persistent severe ARDS | 33 (12.1) | 12 (10.9) | 1 (2.7) | 21 (15.8) | 0.086 |
| **Outcomes** |  |  |  |  |  |
| Usage of vasopressors | 275 (99.3) | 109 (100.0) | 33 (94.3) | 133 (100.0) | 0.016 |
| Vasopressor-free days, days | 0.0 (0.0-12.0) | 0.0 (0.0-16.0) | 2.0 (0.0-22.0) | 0.0 (0.0-4.5) | 0.072 |
| Usage of continuous renal replacement therapy | 114 (41.2) | 47 (43.1) | 10 (28.6) | 57 (42.9) | 0.270 |
| Continuous renal replacement therapy-free days, days | 18.0 (6.0-28.0) | 20.0 (7.0-28.0) | 28.0 (19.0-28.0) | 14.0 (6.0-28.0) | 0.004 |
| Duration of mechanical ventilation among survivors, days | 18.0 (8.3-34.8) | 14.5 (7.3-34.0) | 30.5 (10.8-41.0) | 21.0 (7.3-32.0) | 0.137 |
| Ventilator-free days, days | 0.0 (0.0-6.5) | 0.0 (0.0-15.0) | 0.0 (0.0-13.0) | 0.0 (0.0-1.0) | 0.078 |
| ICU-free days, days | 0.0 (0.0-0.0) | 0.0 (0.0-7.3) | 0.0 (0.0-8.0) | 0.0 (0.0-0.0) | 0.061 |
| ICU-mortality | 147 (52.5) | 49 (44.5) | 9 (24.3) | 89 (66.9) | <0.001 |

*Abbreviations:* n, number; ARDS, acute respiratory distress syndrome; SOFA, sequential organ failure assessment; NA, not available; ECMO, extracorporeal membrane oxygenation; bpm, breaths per minute; PEEP, positive end expiratory pressure; Pplateau, plateau pressure; Pdriving, driving pressure; PaO_2_, partial pressure of arterial oxygen; FiO_2_, fraction of inspired oxygen; PaCO_2_, partial pressure of arterial carbon dioxide: ICU, intensive care unit.

Data are presented as median (interquartile range) or number of patients (%).

Heart condition included congestive heart failure, coronary artery disease, and cardiomyopathies.

Patients, who were intubated outside the ICU, were admitted in the ICU the same day.

Non-invasive mechanical ventilation was delivered via face mask.

Severity of ARDS was classified according to the Berlin definition.

Administration of steroids was initiated prior to intubation.

Intermediate group includes two patients from Crete, who were transferred to another ICU on the 5^th^ and 9^th^ day following intubation, respectively. These patients were considered alive at day 28 following intubation. Persistent severe ARDS group includes 10 patients who were not alive on the second day following intubation.

ICU-mortality of patients with rapidly improving ARDS was 50% (1 of 2) in Athens, 50% (1 of 2) in Crete and 57.1% (4 of 7) in Larissa.

Outcomes other than duration of mechanical ventilation were censored at day 28 following intubation. Patients discharged from ICU with unassisted breathing before 28 days considered to be alive at 28 days without needing vasopressors or continuous renal replacement therapy. Vasopressor-free days, continuous renal replacement therapy-free days, ventilator-free days and ICU-free days were calculated by the number of days in the first 28 days following intubation that a patient was alive and not receiving vasopressors, not receiving continuous renal replacement therapy, not on a ventilator or not in the ICU, respectively.

**e-Figure 1.** **Distribution of scores of Sequential Organ Failure Assessment (SOFA) on the day of intubation among the three study sites.**

**
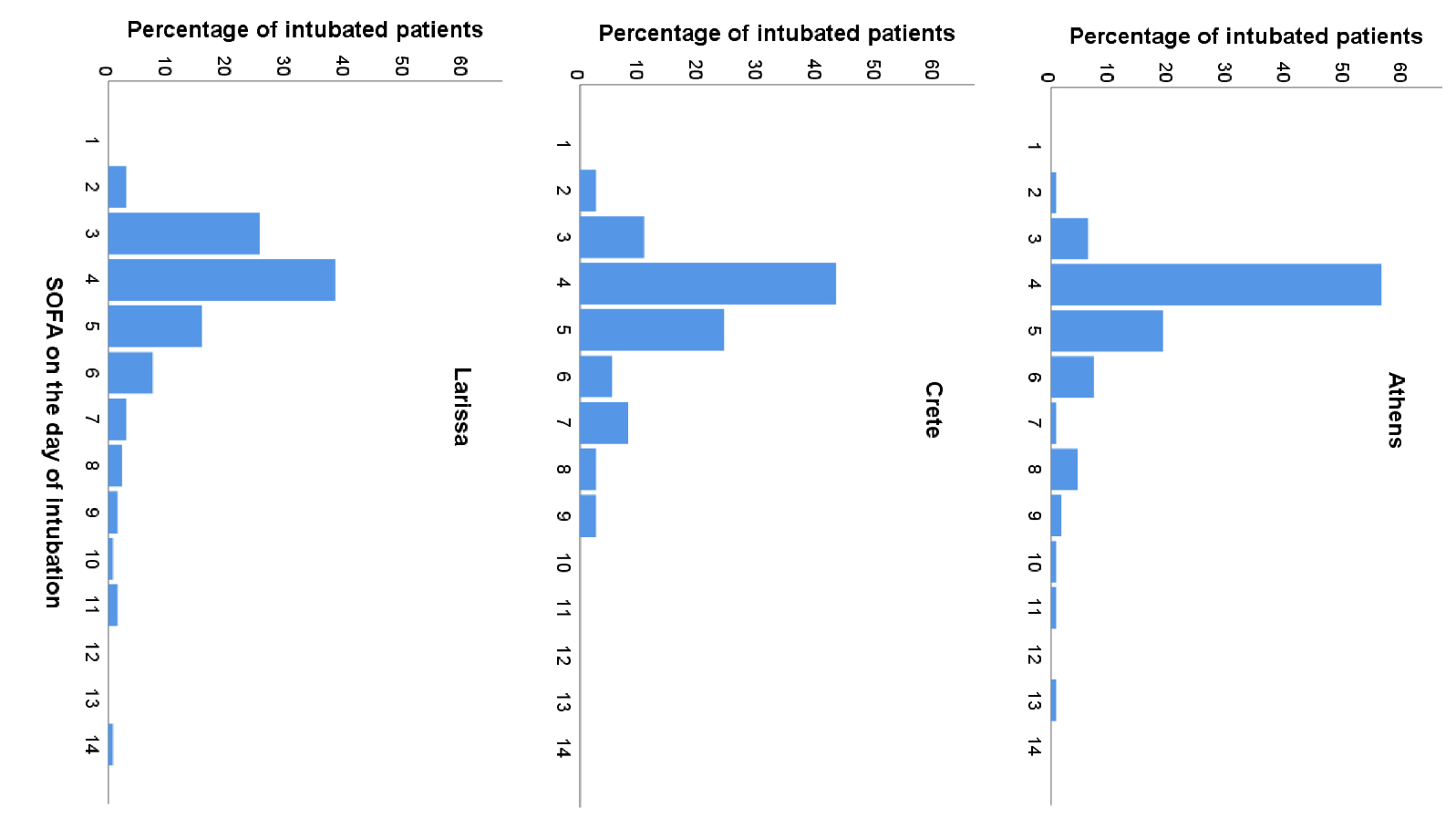
**

**e-Figure 2. Distribution of partial pressure of arterial oxygen to fraction of inspired oxygen ratio (PaO_2_:FiO_2_) values on the day of intubation (calculated after intubation) among the three study sites.**

**
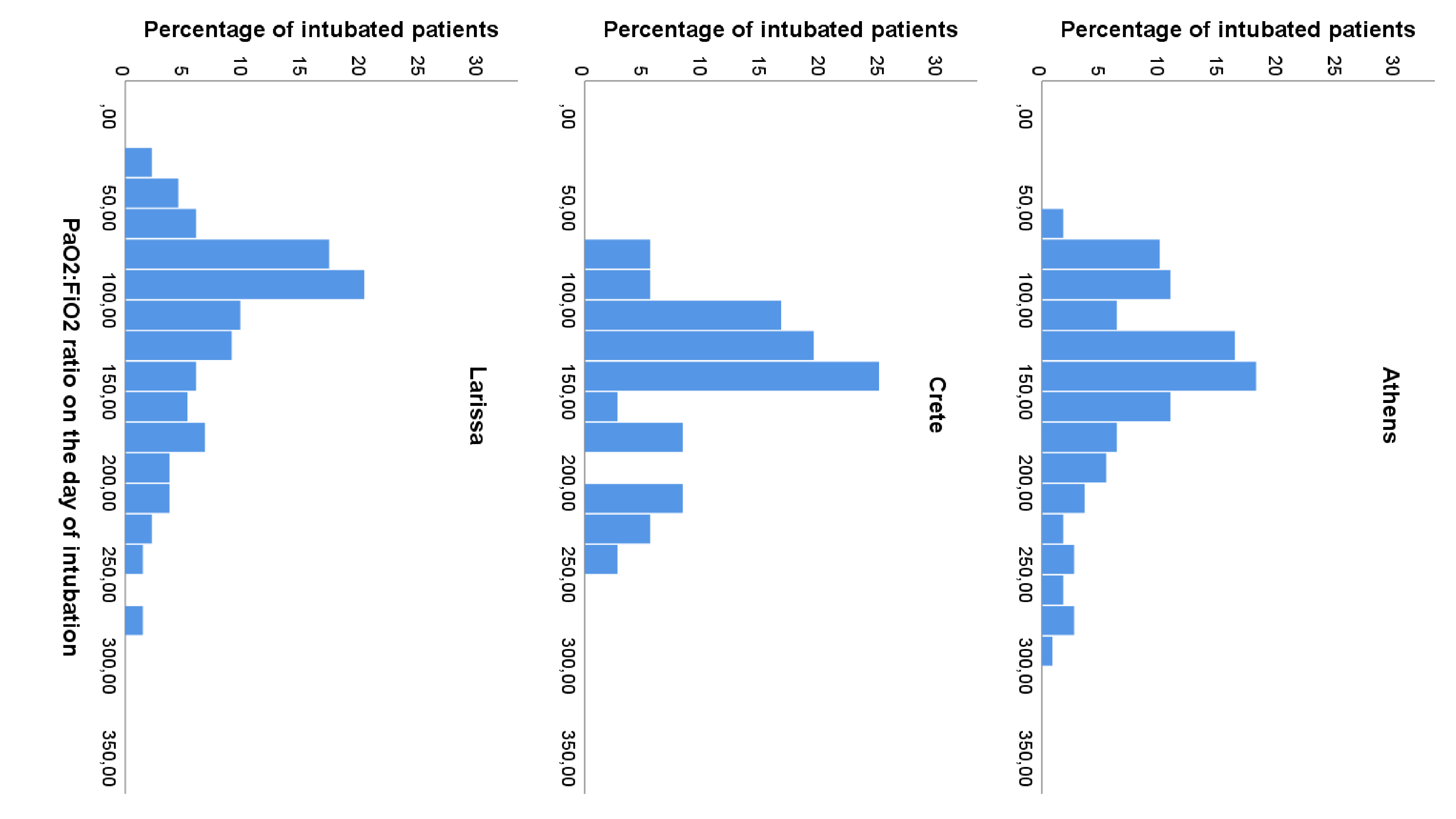
**

| **e-Table 2. Univariable and multiple variable regression analysis to isolate the contribution of age, modified SOFA score on the day of intubation and study site (independent variables) to the ICU-mortality (dependent variable).** | | | | | | |
| --- | --- | --- | --- | --- | --- | --- |
|  | **Univariable analysis** | | | **Multivariable analysis** | | |
|  | **Hazard ratio** | **95% confidence intervals** | **p value** | **Hazard ratio** | **95% confidence intervals** | **p value** |
| Age (increments of 1 year) | 1.042 | 1.025-1.059 | <0.001 | 1.047 | 1.030-1.065 | <0.001 |
| Modified SOFA score on the day of intubation (increments of 1) | 1.197 | 1.102-1.300 | <0.001 | 1.181 | 1.086-1.284 | <0.001 |
| Study site (Athens as reference) |  |  |  |  |  |  |
| Crete | 0.466 | 0.229-0.949 | 0.035 | 0.273 | 0.123-0.608 | 0.001 |
| Larissa | 1.904 | 1.342-2.700 | <0.001 | 1.754 | 1.235-2.491 | 0.002 |

*Abbreviations:* SOFA, sequential organ failure assessment; PaO_2_, partial pressure of arterial oxygen; FiO_2_, fraction of inspired oxygen; ICU, intensive care unit.

Modified SOFA score equals to total SOFA score minus the respiratory component of SOFA score; i.e., it takes into account the coagulation, hepatic, cardiovascular, neurologic and renal components of SOFA.

| **e-Table 3. Univariable and multiple variable regression analysis to isolate the contribution of PaO_2_:FiO_2_, FiO_2_ and driving pressure on the day of intubation (independent variables) to the development of persistent severe ARDS (dependent variable).** | | | | | | |
| --- | --- | --- | --- | --- | --- | --- |
|  | **Univariable analysis** | | | **Multivariable analysis** | | |
|  | **Odds ratio** | **95% confidence intervals** | **p value** | **Odds ratio** | **95% confidence intervals** | **p value** |
| PaO_2_:FiO_2_ (increments of 1) | 0.991 | 0.983-0.999 | 0.026 | 0.999 | 0.988-1.010 | 0.844 |
| FiO_2_ (increments of 0.1) | 1.028 | 1.007-1.050 | 0.010 | 1.025 | 0.996-1.054 | 0.093 |
| Pdriving (increments of 1) | 1.173 | 1.072-1.284 | 0.001 | 1.146 | 1.039-1.264 | 0.006 |

*Abbreviations:* PaO_2_, partial pressure of arterial oxygen; FiO_2_, fraction of inspired oxygen; ARDS, acute respiratory distress syndrome; Pdriving, driving pressure.

| **e-Table 4. Univariable and multiple variable regression analysis to isolate the contribution of age, modified SOFA score on the day of intubation and change in PaO_2_:FiO_2_ from day of intubation to the first day following intubation (independent variables) to the ICU-mortality (dependent variable).** | | | | | | |
| --- | --- | --- | --- | --- | --- | --- |
|  | **Univariable analysis** | | | **Multivariable analysis** | | |
|  | **Hazard ratio** | **95% confidence intervals** | **p value** | **Hazard ratio** | **95% confidence intervals** | **p value** |
| Age (increments of 1 year) | 1.042 | 1.025-1.059 | <0.001 | 1.038 | 1.021-1.055 | <0.001 |
| Modified SOFA score on the day of intubation (increments of 1) | 1.197 | 1.102-1.300 | <0.001 | 1.162 | 1.064-1.270 | 0.001 |
| Change in PaO_2_:FiO_2_ ratio from day of intubation to the first day following intubation | 0.997 | 0.994-1.0 | 0.035 | 0.998 | 0.995-1.001 | 0.181 |

*Abbreviations:* SOFA, sequential organ failure assessment; PaO_2_, partial pressure of arterial oxygen; FiO_2_, fraction of inspired oxygen; ICU, intensive care unit.

Modified SOFA score equals to total SOFA score minus the respiratory component of SOFA score; i.e., it takes into account the coagulation, hepatic, cardiovascular, neurologic and renal components of SOFA.

| **e-Table 5. Characteristics and outcomes of patients hospitalized in Crete with ARDS related to COVID-19 versus influenza.** | | | |
| --- | --- | --- | --- |
|  | **COVID-19 (n=37)** | **Influenza (n=13)** | **p value** |
| Age, years | 73.0 (64.0-78.5) | 58 (50.5-74.5) | 0.049 |
| Female sex | 15 (40.5) | 6 (46.2) | 0.754 |
| Comorbidity | 30 (81.1) | 8 (61.5) | 0.256 |
| Chronic kidney disease | 4 (10.8) | 0 (0) | 0.561 |
| Chronic lung disease | 7 (18.9) | 4 (30.8) | 0.445 |
| Heart condition | 11 (29.7) | 1 (7.7) | 0.147 |
| Diabetes mellitus | 12 (32.4) | 2 (15.4) | 0.303 |
| Malignancy | 5 (13.5) | 1 (7.7) | 1 |
| PaO_2_:FiO_2_ on the day of intubation | 136.5 (114.0-168.3) | 136.0 (126.5-159.0) | 0.237 |
| PaO_2_:FiO_2_ on the first day following intubation | 177.0 (133.5-221.5) | 225.0 (169.0-252.5) | 0.036 |
| ICU-mortality | 9 (24.3) | 3 (23.1) | 1 |
| Rapidly improving ARDS | 2 (5.4) | 2 (15.4) | 0.275 |

*Abbreviations:* ARDS, acute respiratory distress syndrome; COVID-19, new coronavirus disease; n, number; PaO_2_, partial pressure of arterial oxygen; FiO_2_, fraction of inspired oxygen; PaCO_2_, partial pressure of arterial carbon dioxide.

Data are presented as median (interquartile range) or number of patients (%).

Heart condition included congestive heart failure, coronary artery disease, and cardiomyopathies.

ICU-mortality of patients with rapidly improving ARDS was 50% (1 of 2) in COVID-19 and 0% (0 of 2) in influenza group.

| **e-Table 6. Characteristics and outcomes of patients with rapidly improving ARDS due to pneumonia related or not to COVID-19.** | | | |
| --- | --- | --- | --- |
|  | **Pneumonia related to COVID-19 (n=11)** | **Pneumonia not related to COVID-19 (n=70)** | **p value** |
| Age, years | 73.0 (46.0-78.0) | 57.0 (41.3-67.8) | 0.158 |
| Female sex | 5 (45.5) | 39 (55.7) | 0.538 |
| Race |  |  |  |
| Caucasian | 11 (100.0) | 58 (82.9) | 0.203 |
| African | 0 (0.0) | 10 (14.3) | 0.343 |
| Other | 0 (0.0) | 2 (2.9) | 1 |
| Comorbidity | 9 (81.8) | 38 (54.3) | 0.029 |
| Chronic kidney disease | 1 (9.1) | 0 (0) | 0.136 |
| Liver disease | 1 (9.1) | 3 (4.3) | 0.449 |
| Diabetes mellitus | 3 (27.3) | 16 (22.9) | 0.714 |
| Malignancy | 2 (18.2) | 13 (18.6) | 1 |
| Non-pulmonary organ failure | 4 (36.4) | 51 (72.9) | 0.032 |
| Coagulation | 1 (9.1) | 17 (24.3) | 0.441 |
| Hepatic | 0 (0) | 5 (7.1) | 1 |
| Cardiovascular | 1 (9.1) | 42 (60.0) | 0.002 |
| Renal | 3 (27.3) | 16 (22.9) | 0.714 |
| **Lung mechanics on the day of intubation** |  |  |  |
| Tidal volume, mL | 430.0 (380.0-480.0) | 410.0 (350.0-465.0) | 0.386 |
| PEEPtotal, cmH_2_O | 12.0 (10.5-14.0) | 5.0 (5.0-8.0) | <0.001 |
| Pplateau, cmH_2_O | 23.5 (20.0-26.3) | 19.0 (17.0-24.3) | 0.072 |
| Pdriving, cmH_2_O | 12.0 (8.8-13.0) | 12.5 (9.0-15.3) | 0.062 |
| PaO_2_, mmHg | 101.0 (90.0-162.0) | 93.5 (74.0-106.0) | 0.217 |
| PaO_2_:FiO_2_ | 202.0 (162.0-227.0) | 190.0 (148.0-240.0) | 0.894 |
| PaCO_2_, mmHg | 45.0 (39.5-52.7) | 37.0 (34.0-42.0) | 0.014 |
| **Outcomes** |  |  |  |
| PaO_2_:FiO_2_ on the first day following intubation | 353.0 (314.0-368.0) | 279.5 (194.3-325.0) | <0.001 |
| Extubated on the first day following intubation | 0 (0.0) | 7 (10.0) | 0.585 |
| Ventilator-free days, days | 0.0 (0.0-25.0) | 27.0 (23.5-27.0) | 0.006 |
| ICU-free days, days | 0.0 (0.0-22.0) | 24.0 (21.0-26.0) | 0.004 |
| ICU-mortality, n (%) | 6 (54.5) | 9 (12.9) | 0.004 |

*Abbreviations:* ARDS, acute respiratory distress syndrome; COVID-19, new coronavirus disease; n, number; PEEP, positive end expiratory pressure; Pplateau, plateau pressure; Pdriving, driving pressure; PaO_2_, partial pressure of arterial oxygen; FiO_2_, fraction of inspired oxygen; PaCO_2_, partial pressure of arterial carbon dioxide: ICU, intensive care unit.

Data are presented as median (interquartile range) or number of patients (%).

Patients with rapidly improving ARDS due to pneumonia not related to COVID-19 in this Table were derived from the Statins for Acutely Injured Lungs from Sepsis (SAILS) randomized controlled trial performed by the ARDSNet. Out of the patients with ARDS included in the SAILS trial, 529 patients had pneumonia. Of the latter, 70 patients (i.e., 70/529= 13.2%) had rapidly improving ARDS.

All outcomes were censored at day 28 following intubation. Patients discharged from ICU with unassisted breathing before 28 days considered to be alive at 28 days. Ventilator-free days and ICU-free days were calculated by the number of days in the first 28 days following intubation that a patient was alive and not on a ventilator or not in the ICU, respectively.
